# Vitamin E Supplementation and Mitochondria in Experimental and Functional Hyperthyroidism: A Mini-Review

**DOI:** 10.3390/nu11122900

**Published:** 2019-12-01

**Authors:** Gaetana Napolitano, Gianluca Fasciolo, Sergio Di Meo, Paola Venditti

**Affiliations:** 1 Dipartimento di Scienze e Tecnologie, Università degli Studi di Napoli Parthenope, via Acton n. 38, I-0133 Napoli, Italy; gaetana.napolitano@uniparthenope.it; 2 Dipartimento di Biologia, Università di Napoli Federico II, Complesso Universitario Monte Sant’Angelo, Via Cinthia, I-80126 Napoli, Italy; gianluca.fasciolo@unina.it (G.F.); sergio.dimeo@unina.it (S.D.M.)

**Keywords:** mitochondria, vitamin E, hyperthyroidism, cold exposure

## Abstract

Mitochondria are both the main sites of production and the main target of reactive oxygen species (ROS). This can lead to mitochondrial dysfunction with harmful consequences for the cells and the whole organism, resulting in metabolic and neurodegenerative disorders such as type 2 diabetes, obesity, dementia, and aging. To protect themselves from ROS, mitochondria are equipped with an efficient antioxidant system, which includes low-molecular-mass molecules and enzymes able to scavenge ROS or repair the oxidative damage. In the mitochondrial membranes, a major role is played by the lipid-soluble antioxidant vitamin E, which reacts with the peroxyl radicals faster than the molecules of polyunsaturated fatty acids, and in doing so, protects membranes from excessive oxidative damage. In the present review, we summarize the available data concerning the capacity of vitamin E supplementation to protect mitochondria from oxidative damage in hyperthyroidism, a condition that leads to increased mitochondrial ROS production and oxidative damage. Vitamin E supplementation to hyperthyroid animals limits the thyroid hormone-induced increases in mitochondrial ROS and oxidative damage. Moreover, it prevents the reduction of the high functionality components of the mitochondrial population induced by hyperthyroidism, thus preserving cell function.

## 1. Introduction

Aerobic organisms obtain energy required for maintaining their steady-state condition from their highly efficient bioenergetic apparatus, which is dependent on oxygen. Oxygen, however, is a double-edged sword because it gives rise to radicals and other reactive oxygen species (ROS) able to oxidize all biological macromolecules, damaging aerobic cells. Cellular ROS are by-products of most physiological processes involving oxygen, and the mitochondrial electron transport chain is considered their main source [1,2]. The superoxide (O_2_^−^), originating from the univalent reduction of oxygen due to auto-oxidation of mitochondrial carriers [3], is the primary ROS. Hydrogen peroxide (H_2_O_2_) originates from the dismutation of superoxide, either spontaneous or catalyzed, by the enzyme superoxide dismutase (SOD). H_2_O_2_ can then react with iron or copper and generate the hydroxyl radical (OH∙), the most reactive oxygen radical.

Studies suggest that potential sites of O_2_^−^ formation are localized at complex I [3,4], III [5], and II [6,7]; however, there are no certainties relating the exact sites of O_2_^−^ formation and their in vivo contribution [6,7].

Mitochondrial ROS production rate is dependent on physiological or pathological conditions. In any case, only a portion of the formed ROS reaches the cytosol because part of them is removed owing to their reaction with mitochondrial lipids, proteins, and DNA, inducing mitochondrial alterations. Part of the ROS is also removed by the efficient antioxidant system of which the mitochondria are equipped, allowing them not only to neutralize the species they produce but also those produced by other cellular sources [8].

Therefore, when ROS generation by mitochondria and other cellular sites increases, overwhelming the capacity of the mitochondrial antioxidant systems, components of the respiratory chain and enzymes of the Krebs cycle may be deactivated. This can lead to mitochondrial dysfunction, which can have harmful consequences for the cells and the whole organism, resulting in metabolic and neurodegenerative disorders including type 2 diabetes, obesity, dementia, and aging [9].

The mitochondrial antioxidant defense system includes low-molecular-mass antioxidants and a battery of enzymes capable of scavenging ROS or repairing the damage they cause to the biological molecules.

A major site of oxidative damage is the phospholipid bilayer of bio-membranes, with formation of peroxyl radicals and activation of peroxidative cascade [9].

The main antioxidant in the mitochondrial membranes is the lipid-soluble vitamin E that can react with the peroxyl radicals faster than the molecules of polyunsaturated fatty acids [10], thus protecting mitochondrial membranes from excessive oxidative damage.

Vitamin E supplementation can increase the vitamin content in mitochondria, protecting them from the dysfunction due to increased oxidative damage. In the present review, we summarize the available data concerning the capacity of vitamin E supplementation to protect mitochondria from oxidative damage in hyperthyroidism, a condition characterized by oxidative stress.

## 2. Vitamin E

The term vitamin E indicates eight different molecules, synthesized only in the plastids of the photosynthetic organisms, characterized by a hydrophobic isoprenoid tail, and a chromanol head. The tail contains three double bonds in the tocotrienols and is saturated in the tocopherols. Both tocopherols and tocotrienols are distinguished in four different isoforms that differ in terms of the number and the position of the methyl groups on the chromanol heads [11].

In the plants, the α-isoform of tocopherols (α-T) is the major isoform found in leaves, the γ-isoform (γ-T) is the major form in seeds, whereas β- and δ-tocopherols (β-T and δ-T) are much less abundant. Tocotrienols (α, β, δ, and γ-T3) occur mainly in cereals and are less widespread.

### 2.1. Vitamin E Antioxidant Capacity

The antioxidant property of vitamin E is due first to its capacity to deactivate oxygen singlet (_1_O^2^) by quenching. In the study of resonance energy transfer, it has been shown that one molecule of α-T can deactivate up to 120 _1_O^2^ molecules before its degradation [12]. Tocopherols can also act as potent chain-breaking antioxidants chemically scavenging _1_O^2^ and lipid peroxyl radicals. The first action irreversibly produces quinones and epoxides, whereas the second one results in the formation of the tocopheroxyl radical [13]. This radical can be recycled back to α-tocopherol by ascorbate. Therefore, the tocopherols can trap propagating radical intermediates that are produced during lipid peroxidation and arrest the chain reactions.

Tocopherols may also stabilize membrane structures by interacting with polyunsaturated fatty acyl chains (non-antioxidant functions) [14,15], and are involved in carbohydrate metabolism [16] and cell proliferation [14] in plants.

Generally, it is assumed that the major biological role of vitamin E is to protect polyunsaturated fatty acids (PUFA) and other components of cell membranes and low-density lipoprotein (LDL) from oxidation by free radicals [17]. It is also reported that vitamin E accounts for most of the lipid-soluble antioxidant activity in mammalian tissues and plasma, and its deficiency has been associated with an elevated risk of atherosclerosis and other degenerative diseases. According to its antioxidant activity, vitamin E reduces the inflammatory processes by limiting the generation of ROS and their damaging effects [18]. In fact, Calder demonstrated that α-tocopherol decreases the release of pro-inflammatory cytokines and nuclear factor kappa-light-chain-enhancer of activated B cells (NFkB) activation [18]. Although it is currently believed that increases of α-tocopherol in the diet contribute to a decreased risk of chronic diseases [11], dietary supplementations with α-tocopherol alone displayed only a modest protective effect. Some authors proposed that high vitamin E intake can reduce the risk of some cancer forms, such as prostate [19] and colon [20] cancers. Although α-tocopherol is the biologically most active form of vitamin E in the human body [21], the presence in the diet of both δ- and γ- in addition to α-tocopherol seems to be imperative in achieving optimal biological effects [22].

### 2.2. Vitamin E Metabolism and Subcellular Distribution

In animals, vitamin E is acquired through the ingestion of vitamin E rich foods, among which oils, nuts, seeds, and wholegrain cereals are included. α-Tocopherol represents by far the most common form of vitamin E in human tissues [23] and its serum concentration is weakly associated with fruit and vegetable consumption [24]. However, the quantity and quality of the fat present in an individual’s diet largely influences the vitamin E absorption [11]. The mechanisms of vitamin E intestinal absorption are only partially understood. Data obtained in recent years, show that its intestinal absorption is mediated, at least in part, by cholesterol membrane transporters including the scavenger receptor class B type I (SR-BI), cluster differentiation 36 molecule (CD36), Nieman–Pick-C1/like 1 (NPC1L1), and ATP-binding cassettes A1 and G1 (ABCA1 and ABCG1) [25].

From the intestine, dietary tocopherols and tocotrienols, incorporated in chylomicron particles with triacylglycerol, phospholipids and cholesterol [26,27,28], are transported to the peripheral tissues, including muscle, bone marrow, adipose, skin, and possibly brain via the lymphatic system. Chylomicron-associated tissue uptake of vitamin E takes place through a lipoprotein receptor-mediated process, which is not well understood [26,27,29]. This process allows for the accumulation of γ-T in human skin, fat, and muscle, where unexpectedly high concentrations of γ-T are observed, in contrast to its low levels in the plasma [30]. The resulting chylomicron remnants are subsequently taken up by the liver. The α-T enters in the hepatocytes by endocytosis. It reaches the late endosomal compartment where it binds to an α-tocopherol transfer protein (αTTP), present on the outer leaflet of the endosomal membrane. This interaction facilitates the transport of α-T to the plasma membrane, where its binding to the resident phosphatidylinositol 4,5-bisphosphate induces a conformational change, resulting in the release of α-T and its incorporation into the membrane [31,32,33]. The subsequent bond with ABCA1 allows α-T to exit the cells, be incorporated into lipoproteins, and be delivered to extrahepatic tissues. αTTP repeats the cycle, translocating itself to the endosomal compartment [31]. α-TTP is largely affine to α-T (100%) and has a much lower affinity toward to β-T, γ-T, and δ-T (50%, 10%–30%, or 1%, respectively) [34]. Unlike α-T, which is bound and thus protected by α-TTP, large portions of non-α-T forms of vitamin E are catabolized and at once oxidized to quinones. Vitamin E can be conjugated with glucuronic acid and excreted in the feces [17]. It is also localized in the membrane of organelles such as endoplasmic reticulum, mitochondria, and peroxisomes [35,36,37,38,39]. In the past, it was proposed that a cytosolic binding protein could be involved in the distribution of vitamin E to mitochondria [40]. However, the problem concerning vitamin E distribution among the membrane of cellular organelles has been only recently investigated by Irias-Mata and colleagues [41]. Using cultured hepatocytes with or without a stable expression of αTTP [41], they found that αTTP is not involved in mitochondrial targeting. In fact, α-T was significantly correlated with the mitochondrial cellular fraction irrespective of αTTP expression, and meanwhile γ-T, α-tocotrienol (α-T3), and γ-tocotrienol (γ-T3) were not [41]. The authors proposed diffusion as the main driving force of the distribution of vitamin E isoforms within the cell [41]. Moreover, they found that the metabolites of γ-T, α-T3, and γ-T3, rather than the parent compounds, localize in mitochondria, whereas the metabolites of β-T, γ-T, and δ-T localize in peroxisomes [41]. This observation concurs with the function of these organelles in the metabolism of vitamin E in general and the preferential metabolism of the non-α-T isoforms. Thus, the initial ω-hydroxylation of the parent vitamin E occurs in the endoplasmic reticulum. Then, this alcohol metabolite is ω-oxidized in the peroxisomes and the product is β-oxidized in the mitochondria to ultimately yield the sidechain-shortened carboxyethyl hydroxychromanol metabolites [42,43].

## 3. Vitamin E and Mitochondria

Few data exist on the effect of in vivo vitamin E supplementation in mitochondria. Some information is available on the changes in mitochondrial vitamin E levels from studies on skeletal muscle of livestock, performed to improve food muscle resistance to lipid oxidation [44]. Such studies showed that increased vitamin E concentration seems to be able to protect livestock muscle from oxidation [45,46,47]. In addition, vitamin E preferentially incorporates into the plasma membrane and the membranes of microsomes and mitochondria [44]. Although the mechanism of vitamin E delivery to cellular organelles has not been defined, its preferential distribution to mitochondria concurs with the observation of the existence of a cytosolic binding protein able to transfer the vitamin from liposomes to mitochondria [40].

Even fewer data are available regarding the effects of vitamin E supplementation on mitochondria isolated from animals with increased oxidative stress. Some of these relate to the effects of vitamin E on mitochondria from hyperthyroid animals.

### 3.1. Effects of Vitamin E Supplementation on Mitochondria in Experimental Hyperthyroidism

Thyroid hormones, of which the triiodothyronine is considered the major active form, are the principal regulators of vertebrate metabolic rate. They perform many physiological functions, influencing the growth, development, and metabolism of vertebrates [48]. The increased plasma level of thyroid hormones is associated with the onset of oxidative stress in the target tissues of the hormone due to the alteration in the balance between pro-oxidants and antioxidants [49]. The mitochondria release ROS at a higher rate in the hyperthyroid animals than in the euthyroid animals. Swaroop and Ramasarma [50] first reported that treatment with T_4_, in a single dose or three doses for three consecutive days, increases mitochondrial H_2_O_2_ generation in rat liver. Subsequently, Fernández and Videla found that daily doses of T_3_ for three consecutive days enhance the rate of superoxide radical generation in the submitochondrial particles of rat liver supported by succinate or reduced nicotinamide adenine dinucleotide (NADH) [51]. Longer T_3_ treatments confirmed that the thyroid hormone also affects mitochondrial H_2_O_2_ release on other target tissues of the hormone. In fact, 10 days of administering T_3_ (10 μg/100 g body weight) to hypothyroid animals enhances the rate of H_2_O_2_ release by the liver [52], skeletal muscle (gastrocnemius) [53], and heart [54] mitochondria. These experiments were conducted using succinate and a mix of pyruvate and malate as substrates linked to complex II and I, respectively. In the hyperthyroid rats, the H_2_O_2_ release rate increased during both basal (state 4) and adenosine diphosphate (ADP)-stimulated (state 3) oxygen consumption in liver and heart mitochondria, but only during basal respiration in muscle mitochondria. The same thyroid hormone treatment increases the oxidative damage to lipids and proteins and the susceptibility to oxidative damage in the liver, heart, and muscle mitochondria [55]. In general, the high susceptibility of mitochondria to ROS depends on the fact that they are the major sites of ROS production, but also on their high content of Fe^2+^ complexes of high and low molecular weight. It is known that these complexes favor the oxidative damage to mitochondrial membrane lipids [56,57]. In hyperthyroid mitochondria, the susceptibility to ROS is reinforced due to the thyroid hormone-induced increase in the content of the respiratory chain components including the autoxidizable ones [49].

The increased oxidative damage is also responsible for the induction of mitochondrial nonspecific pores that determine the mitochondrial permeability transition, that is, a sudden increase in the permeability of the inner mitochondrial membrane to molecules of mass up to 1500 Da [58,59,60,61]. These pores, defined as mitochondrial permeability transition pores (MPTP), occur when mitochondria are overloaded with calcium. The sensitivity of the MPTP to calcium is enhanced under oxidative stress conditions, adenine nucleotide depletion, high phosphate concentrations, or membrane depolarization [62]. MPTP opening is responsible for the swelling of the mitochondrial matrix, the collapse of membrane potential, and the uncoupling of oxidative phosphorylation [63]. The ROS induction of MPTP [64] is likely due to the oxidation of membrane protein thiols unmasked by matrix Ca^2+^ [65].

T_3_ treatment is associated with the increased mitochondrial susceptibility to Ca^2+^-induced inner membrane permeabilization, as shown by the increased swelling and fall of inner membrane potential [56]. This can be due to the enhanced mitochondrial oxidative damage. Interestingly, the increased oxidative damage and susceptibility to the oxidant and the calcium-induced swelling are associated with a reduction of total antioxidant capacities but not to changes in the vitamin E content in hyperthyroid mitochondria from liver, heart, and muscle [56]. Ten daily intramuscular injections of 20 mg/100g bodyweight of α-tocopherol increased the content of vitamin E in hyperthyroid rat liver mitochondria [65]. Vitamin E treatment was able to reduce the thyroid hormone-induced increase in lipid oxidative damage and prevent the reduction in total antioxidant capacities [65].

The changes in mitochondrial oxidative damage can influence the turnover of the mitochondrial population. Mitochondria can be resolved by differential centrifugation in subpopulations that differ for their functional characteristics and relative amount. In rat liver, the lighter fractions have lower respiratory capacity and may represent the precursors of heavier fractions. These heavier fractions are characterized by a higher respiratory capacity [66], ROS release, and susceptibility to oxidants [67], in addition to a lower antioxidant capacity. The separation of mitochondrial population of euthyroid rat liver in three fractions (at 1000, 3000, and 10,000 g, defined M_1_, M_3_, and M_10_, respectively) revealed that M_1_ is the most abundant and M_10_ the least abundant. The M_1_ fraction also has the greatest susceptibility to the swelling induced by calcium [68]. It has been proposed that the formation of MPTP may activate the elimination of damaged mitochondria, protecting the cells from these ROS overproducing mitochondria [69]. MPTP seems to be involved in the onset of mitophagy [70], a dynamic process of mitochondrial elimination [71] that in mammals depends on the phosphatase and tensin homolog (PTEN)-induced putative protein kinase 1 (PINK1) and E3 ubiquitin ligase (Parkin) [72]. The MTPT determines a decrease in the mitochondrial membrane potential, which in turn stabilizes PINK1 on the mitochondrial outer membrane. This is followed by the migration of the cytoplasmic Parkin to the mitochondrial outer membrane. Parkin promotes the ubiquitination of damaged mitochondria and mitophagy. It should be made clear that several distinct forms of mitophagy exist, depending on the type of stimuli and the requirement of autophagic machinery [73].

In the hyperthyroid liver, the relative amount of the M_1_ fraction decreases and this change seems to be due to the increased susceptibility to in vitro calcium-induced swelling [68]. Thyroid hormone also activates mitochondrial proliferation by inducing the transcription of genes encoding for components of the respiratory chain, including those that are autoxidizable [49]. Thus, it couples the induction of mitochondrial protein synthesis with their increased degradation.

Vitamin E treatment prevents the thyroid hormone-induced changes in the distribution of the mitochondrial population among their fractions [65]. This is due to the capacity of vitamin E to reduce the degradation of the M_1_ fraction increasing its antioxidant defenses. In hyperthyroid vitamin E-treated rats, the hepatic distribution of the mitochondrial proteins among the different fractions tend to return to euthyroid values. However, vitamin E does not modify the thyroid hormone-induced increase in the content of the respiratory chain components [65].

### 3.2. Effects of Vitamin E Supplementation on Mitochondria in Functional Hyperthyroidism

In homeothermic animals, the survival to the exposure to low environmental temperatures requires the production of extra heat through processes in which the thyroid hormones play a key role [74]. Following cold exposure, triiodothyronine plasma levels increase, and a condition called functional hyperthyroidism takes place [74]. As a side effect of the metabolic response to cold, oxidative damage develops in cold-exposed animals [75,76,77,78]. It was demonstrated that 10 days of cold exposure increases the levels of oxidative damage to lipids and proteins and the ROS releasing rate by mitochondria isolated from the liver [79] and muscle [80]. Interestingly, cold exposure increases α-tocopherol levels in the hepatic mitochondria [79], and this suggests a mobilization of endogenous tocopherol reserve and/or its intense assimilation from food under the effect of cold, without, however, preventing oxidative damage. Vitamin E supplementation to cold-exposed animals reduces the levels of oxidative damage in both liver [79] and muscle mitochondria [80]. Moreover, vitamin E supplementation potentiates the cold-induced increase in mitochondrial oxygen consumption during the ADP-stimulated respiration [79,80]. This observation could be explained by admitting that in cold exposed animals the mitochondrial respiration is lower than their equipment of electron carriers should allow, and that vitamin E unmasks this phenomenon by protecting chain respiratory components against oxidative damage-linked alterations.

Vitamin E supplementation also attenuates the cold-induced increase in the rate of mitochondrial hydrogen peroxide release in liver and muscle [79,80]. The reduced hydrogen peroxide production could be due to the capacity of vitamin E to react with the precursor of the hydrogen peroxide, the superoxide radical, which can be scavenged not only by α-tocopherol [81] but also by the α-tocopheroxyl radical [82]. This is also confirmed by the observation that the H_2_O_2_ generation in submitochondrial particles obtained from mouse tissues is lowered by vitamin E supplementation in a dose-dependent fashion [83]. Moreover, because vitamin E can regulate the expression of several genes [84], it is conceivable that the observed effects on the H_2_O_2_ generation can depend on changes in the content of autoxidizable mitochondrial electron carriers or on the enzymes that metabolize the H_2_O_2_. The analysis of the effects of electron chain inhibitors on the mitochondrial releasing rate of H_2_O_2_, suggests that vitamin E could affect the content of autoxidizable carriers. It is worth noting that the block of the respiratory chain with an inhibitor completely reduces the electron carriers localized on the substrate side. Therefore, if the ROS generator is located on this side, the H_2_O_2_ production also increases and will only depend on the concentration of the autoxidizable carriers. In liver mitochondria, treatment of cold-exposed rats with vitamin E reduces the cold-induced increase of H_2_O_2_ releasing rates measured (1) in the presence of succinate, rotenone, and antimycin A, and (2) in the presence of pyruvate plus malate and rotenone [79]. The first combination provides information on the content of the autoxidizable carrier located at complex III, the latter one on that located at complex I. Thus, the reduction of the ROS releasing rate can depend on capacity for vitamin E to decrease the cold-induced increase of mitochondrial components.

Further information on the effects of vitamin E supplementation on mitochondria has been supplied by studying the characteristics of the rat liver mitochondrial subpopulations [85]. Cold exposure reduces the relative content of the M_1_ fraction, which exhibits the highest oxidative capacities. This, however, does not avoid the cold-induced increase in oxidative capacities of both the tissue and the whole mitochondrial population [85]. Vitamin E does not affect the cold-induced increase in the liver content of mitochondrial proteins but prevents the changes in the distribution of mitochondrial proteins among the fractions M_1_ and M_3_. The reduction in the levels of oxidative stress markers, observed also in M_1_, confirms that the fall in the content of this fraction depends on an increased oxidative damage and that the vitamin E, by reducing the damage, reduces its loss [85]. Vitamin E also lowers the H_2_O_2_ releasing rate, the calcium-induced swelling, and the fall in mitochondrial membrane potential in all mitochondrial fractions [85]. In conclusion, vitamin E is able to also preserve the mitochondrial function in functional hyperthyroidism, offering protection to the components of the electron chain against the oxidative damage.

### 3.3. Vitamin E Protects Heart Mitochondria from Ischemia-Reperfusion-Induced Damage in Cold Exposed Rats

Information on the capacity of a tissue, and its mitochondria, to oppose to oxidative challenges can be obtained using ischemia-reperfusion (I-R) as a model of oxidative stress. The reoxygenation of ischemic tissue can restore organ function but also leads to tissue/organ damage that is due to ROS production and oxidative stress [86]. Mitochondria play a critical role in the I-R-induced damage [87]. When the rat heart is subjected to I-R, the increased oxidative stress is associated with the increased mitochondrial ROS release, which, in turn, is associated with the decrease in the rate of ADP-stimulated respiration [88]. These changes increase when the duration of ischemia increases [88]. The heart functional recovery from ischemia is reduced in hyperthyroid rats. Studies show that both T_3_ administration [89] and cold exposure [90] reduce the inotropic recovery that is associated with higher levels of oxidative stress markers than those found in the euthyroid heart. Vitamin E administration ameliorates the heart functional recovery after I-R in both experimental [89] and functional [90] hyperthyroidism, lowering the oxidative damage. Evidence was also obtained that mitochondria are involved in the tissue derangement of the hearts from cold-exposed animals. These mitochondria display a faster production of ROS, which causes mitochondrial oxidative damage and functional decline that parallels the tissue dysfunction. Furthermore, vitamin E-linked improvement of heart function is associated with lower oxidative damage and a restored functionality of mitochondria [90]. Other information on the protection offered by vitamin E against I-R of hearts from cold-exposed rats came from the study of the content and the characteristics of two mitochondrial fractions (heavy and light) obtained by differential centrifugation [90]. The heavy fraction displayed higher respiratory capacity and ROS production than the light fraction. However, both mitochondrial fractions showed higher ROS production and susceptibility to the in vitro Ca^2+^-induced swelling with respect to the fractions obtained from euthyroid rats. Vitamin E administration to cold-exposed animals reduced ROS release and the sensitivity of the mitochondrial fractions when challenged with Ca^2+^. The increased sensitivity to calcium-induced swelling of mitochondria from hearts of cold-exposed rats after I-R was also responsible for the reduction in the relative content of the heavy fraction with the corresponding increase in the light fraction. Vitamin E administration prevented the changes in the relative content of the two fractions. The changes in the composition of the mitochondrial population elicited by vitamin E suggest that the antioxidant treatment reduces mitochondrial dysfunction in re-perfused hearts from cold-exposed animals, limiting the loss of the heavy mitochondria, which are those with a higher respiratory capacity. Therefore, vitamin E, limiting the cold-induced peroxidation of the membrane lipids and reducing ROS production and susceptibility to Ca^2^^+^ load, prevents mitochondrial injury, thus preserving heart functional recovery following I-R [90].

## 4. Conclusions and Perspective

**Figure 1:**
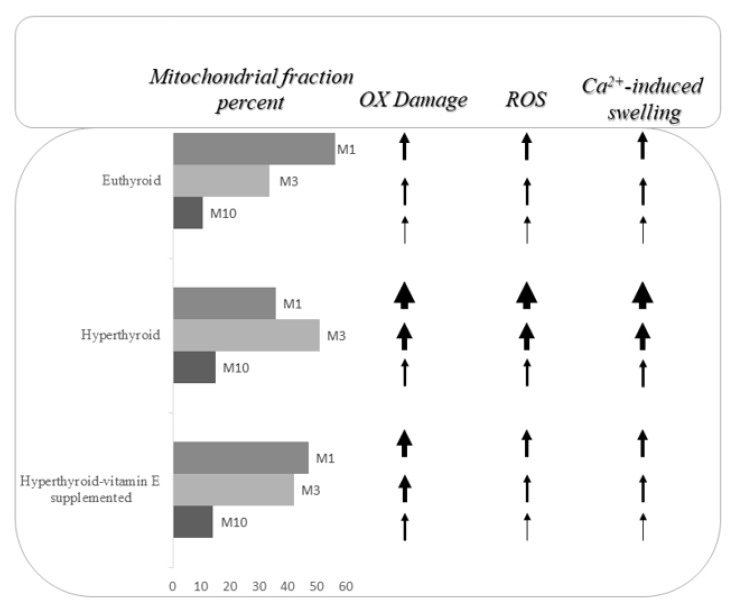
Image representative of the vitamin E supplementation effects on liver mitochondrial fractions obtained at 1000, 3000, and 10,000 g. OX: oxidative damage. The different thickness of the arrows indicates the different sizes of the changes. ROS: reactive oxygen species.

In conclusion, even though it is not yet clear how vitamin E reaches the mitochondrial membranes in cells, it is evident that its administration can prevent, or at least reduce, mitochondrial oxidative dysfunction. Some experimental evidence suggest that the protection offered by vitamin E reduces the loss of the mitochondria, which have the highest functionality in the cell (Figure 1). This protection could be mediated by the capacity of vitamin E to scavenge the ROS, but it is not possible to ignore the fact that it acts also through the modulation of the characteristics of the mitochondrial population that make it more sensitive to oxidative attack.
